# A Fatal Case of Primary Basaloid Squamous Cell Carcinoma in the Intrahepatic Bile Ducts

**DOI:** 10.1155/2014/410849

**Published:** 2014-10-07

**Authors:** Johan Kirkegaard, Mie Grunnet, Jane Preuss Hasselby

**Affiliations:** ^1^The Department of Gastroenterology, Rigshospitalet, Blegdamsvej 9, 2100 Copenhagen Ø, Denmark; ^2^The Department of Oncology, Rigshospitalet, Blegdamsvej 9, 2100 Copenhagen Ø, Denmark; ^3^The Department of Pathology, Rigshospitalet, Blegdamsvej 9, 2100 Copenhagen Ø, Denmark

## Abstract

Primary squamous cholangiocellular carcinomas are very rare. We describe a case of a 67-year-old man, who underwent chemotherapy and surgery for a right-sided liver tumor with an unusual presentation of metastasis to a lymph node in the left armpit. The patient was asymptomatic at the time of diagnosis but expired 20 months after surgery with epidural, lung, and spine metastasis. In addition to the unusual clinical presentation, the diagnosis of the liver tumor was that of a primary basaloid squamous cell carcinoma of the intrahepatic bile ducts, an entity with only one previous report in the literature.

## 1. Introduction 

Carcinoma of the bile ducts is a relatively rare disease with an incidence of 2–6 cases/100.000 people each year in the western world [1]. It has a grave prognosis with a one-year survival rate of 53% [2]. Cholangiocellular carcinoma (CC) is most common among women and the average age of onset is 50 years [3]. It can present in both intra- and extrahepatic biliary ducts, with the intrahepatic form (ICC) being the second most frequent primary liver tumor, accounting for 15% of all liver cancers worldwide [4]. The majority of ICCs is adenocarcinomas [4], whereas pure squamous cell carcinomas are very rare. Only one previous case of primary basaloid squamous cell carcinoma (BSCC) of the intrahepatic bile ducts has been reported [5], whereas metastatic basaloid squamous cellular carcinomas in the liver are well known and more frequently seen [6, 7]. Several predisposing factors of ICC have been reported including hepatolithiasis, Caroli's syndrome, congenital hepatic fibrosis [8], choledochal cysts, biliary tract stone disease, alcoholic liver disease, and cholangitis [9, 10]. The only curative treatment for ICC is surgical resection, but less than 20% of these patients are resectable at the time of diagnosis [11]. Chemotherapy can be given in a noncurative setting, prolonging the overall survival from 8.1–11.7 months [12]. We herein present a very rare case of primary ICC with a basaloid squamous morphology.

## 2. Clinical History

A 67-year-old man was referred to our hospital under the diagnosis “unknown primary tumor.” At first the patient was examined at the referring hospital after being conservatively treated for cholecystitis. An ultrasound (US) scan was performed, showing multiple tumors in the liver and a process in the left armpit.

Following the US-finding a fludeoxyglucose positron emission tomography (FDG-PET) scan, a computer tomography (CT) scan, a sigmoidoscopy, and a gastroscopy were done. The examinations revealed a pathologic fludeoxyglucose (FDG) uptake in the left armpit and three larger hypodense FDG uptake areas in the right lobe of the liver. Apart from the left armpit, no other extrahepatic activity was detected.

Biopsies revealed a basaloid squamous cell carcinoma (BSCC) in the liver, and in the armpit a lymph node with metastatic BSCC was found. Apart from the described incidence of cholecystitis, there were no known dispositions of biliary diseases. At admission the patient was asymptomatic and a physical examination revealed an adequate state of health. The patient's past medical history was unremarkable. Laboratory studies, including tumor markers, were normal except for increased liver parameters (plasma alanine aminotransferase: 81 u/L (10–70), plasma aspartate transaminase: 53 u/L (15–45), and alkaline phosphatase: 127 u/L (35–105)).

The patient received eight series of platin based chemotherapy (Taxol 280 mg iv. and Carboplatin 816 mg iv.), and the following posttreatment CT-scan showed partial response (RECIST 1.1) with reduced size of the tumors in the liver and total regression of the tumor in the armpit.

The remaining tumors left in the liver were hereafter removed surgically with a nonradical right-sided hepatectomy and a cholecystectomy.

Postoperative complications arose in form of bile leakage, reoperation, infection with* Candida albicans* and liver insufficiency. The patient was discharged from the hospital 6 weeks after surgery. At 3-month followup the patient was in a good general condition, but a control CT-scan showed tumor recurrence in the liver. The patient declined repetitive chemotherapy. At 9-month followup, the patient was still alive with a performance status of 0 (Zubrod score/WHO). A new CT-scan showed progression of the tumors in the liver, tumor recurrence in the left armpit, and metastasis to the spine with epidural invasion at the level of T12. New biopsies taken from the liver and armpit showed BSCC, identical to earlier biopsies. Radiation therapy with the RapidArc technic at a dose of 3 Grays × 10 (30 m^2^/s^2^) against the spinal metastasis was initiated and followed by systemic chemotherapy: Gemcitabine and Oxaliplatin given i.v. on days 1 and 15 at doses of 1000 mg/m^2^ at 10 mg/min/m^2^ and 60 mg/m^2^, respectively, in combination with oral Capecitabine on days 1–7 and 15–21 at a dose of 1000 mg/m^2^ b.i.d. The patient did not respond to the first series of chemotherapy and a posttreatment magnetic resonance (MR) scan showed progression of metastasis in liver, spine, and additionally metastasis in the lungs.

The patient expired 1 year and 8 months after the primary surgical resection.

## 3. Materials and Methods

The biopsies and the resected specimens were formalin fixed. Blocks of tumor tissue were sampled from the resected specimen and both biopsies and the sampled tumor blocks were paraffin embedded. 4 *μ*m thin sections for hematoxylin-eosin (HE) staining and immunohistochemistry were cut and mounted on to coated slides. For the immunohistochemical stainings, including pretreatment, the BenchMark ULTRA (Ventana) was used.

The primary antibodies, dilutions, pretreatment, incubation, and sources are presented in Table 1.

## 4. Molecular Analysis

Genomic DNA was extracted from 4 × 10 *μ*m slices from formalin-fixed, paraffin-embedded (FFPE) tissue blocks. Cells were proteinase K-digested overnight at 56°C and DNA automatically purified using the QIAamp DNA mini kit on a QIAcube system (Qiagen, Hilden, Germany) according to the manufacturer's instructions.

PCR amplification of genomic DNA was carried out in a total volume of 25 *μ*L containing 0.4 *μ*mol/L of each primer, 12.5 *μ*L RedEx PCR master mix (Sigma-Aldrich, Brøndby, Denmark), and 50–100 ng genomic DNA. PCR conditions consisted of initial denaturation at 95°C for 5 min, 35 cycles at 95°C for 30 s, 55°C for 30 s and 72°C for 30 s, and a final extension at 72°C for 10 min. All primers used in this study are listed in Table 2. Pyrosequencing was performed using a PyroMark Q24 instrument (Qiagen) according to manufacturer's instructions. Briefly, the PCR product (25 *μ*L) was bound to streptavidin Sepharose HP (GE Healthcare, Brøndby, Denmark), purified, washed, denatured in 0.2 mol/L NaOH, and washed again. Before pyrosequencing, 0.3 *μ*mol/L pyrosequencing primer was annealed to the purified single-stranded PCR product by heating to 80°C for 2 min. Dispensation and analysis orders for the pyrosequencing reactions are listed in Table 2.

Pyrosequencing assays for* KRAS* were evaluated by dilution series of DNA purified human cancer cell lines HCT116 (G13D), SW-620 (G12V), RPMI-8226 (G12A), and HeLa (*KRAS* wildtype). For the* IDH1* and* IDH2* assays, synthetic plasmids containing target regions or with introduced R132H (*IDH1*) and R172K (*IDH2*) mutations were used for validation. The cut-off values were determined to 10% tumor cells/5% mutant allele for all assays.

## 5. Pathology

**Figure 1:**
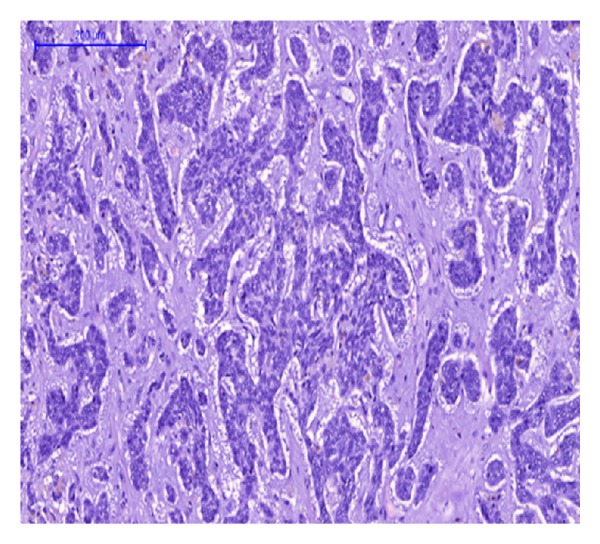
HE-staining. Hepatic BSCC showing tumor infiltration, islands, and strands with surrounding retraction columns.

**Figure 2:**
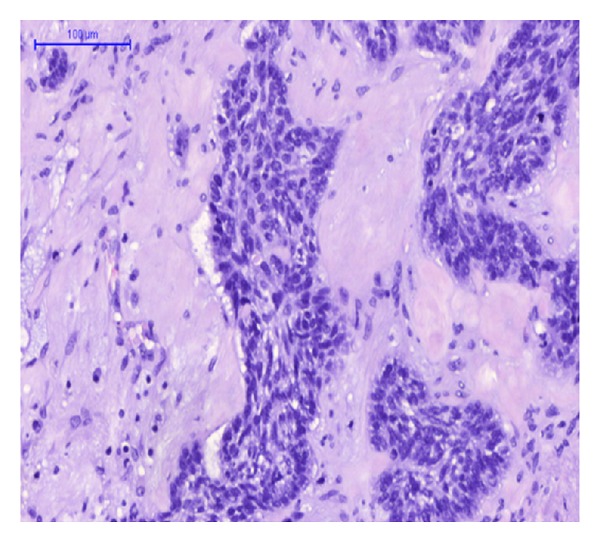
HE-staining. Hepatic BSCC showing basaloid cells with hyperchromatic nuclei and scant cytoplasm. The tumor cells are arranged in solid islands and strands with peripheral palisading.

**Figure 3:**
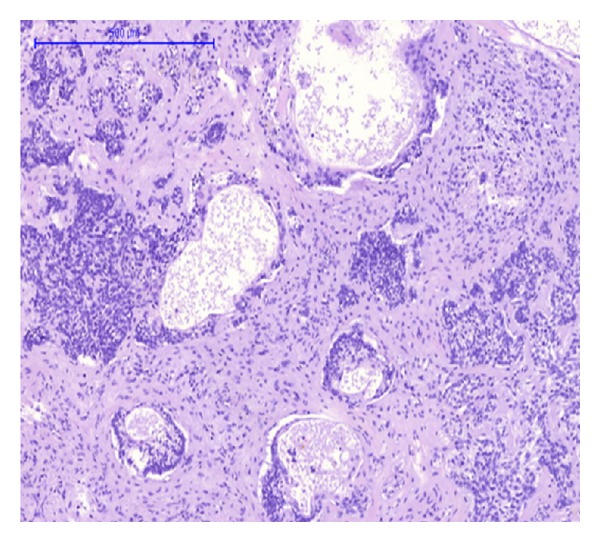
HE-staining. Hepatic bile ducts with transition from metaplastic to dysplastic epithelium and invasion.

**Figure 4:**
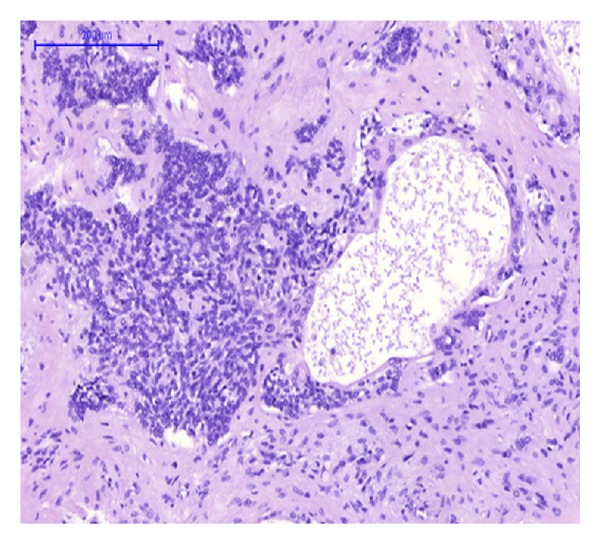
HE-staining. Hepatic bile duct with tumor cells in the lumen, and transition from metaplastic to dysplastic epithelium and invasion.

**Figure 5:**
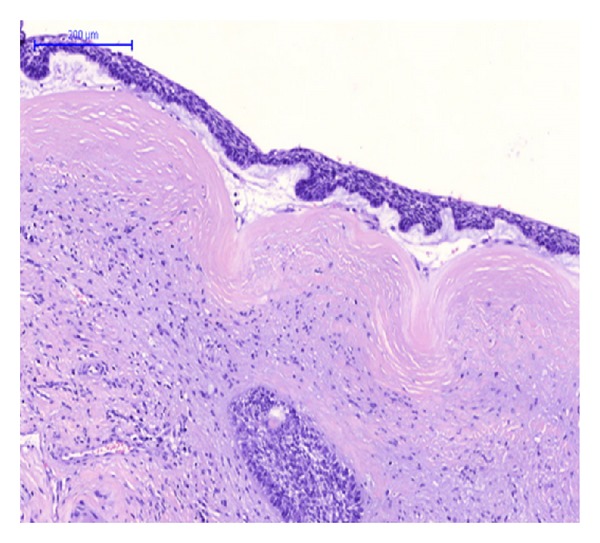
HE-staining. Large hepatic bile duct with dysplastic epithelium.

Gross examination of the main liver specimen revealed several white tumor processes measuring 1–3, 5 cm in diameter. Histologically the tumors consisted of large solid, tumor islands, and strands in a fibrous stroma with some necrosis. Around several of the tumor islands retraction column patterns were found (Figure 1). The tumor cells were relatively uniform with irregular and oval nuclei, with a dense chromatin structure and distinct nucleoli (Figure 2). Several bile ducts were infiltrated by tumor cells, and others showed an obvious transition from well-differentiated columnar epithelium to a metaplastic squamous epithelium and to dysplasia and areas of invasive growth (Figures 3, 4, and 5).

**Figure 6:**
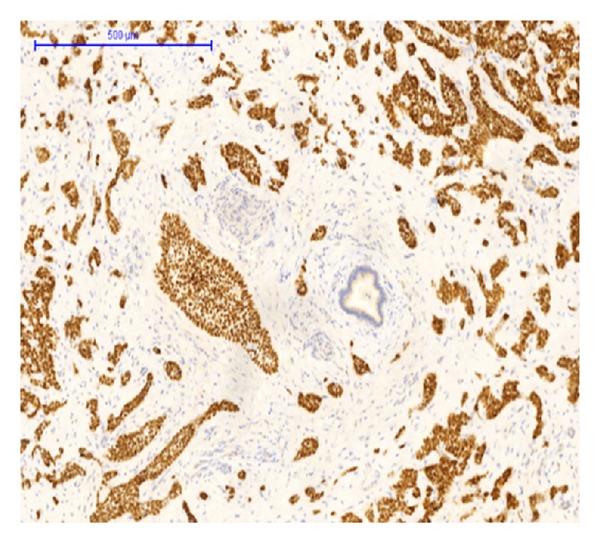
P63 immunohistochemical staining. Hepatic BSCC showing intense staining. Tumor cells are positive, whereas the benign bile duct in the center is negative.

**Figure 7:**
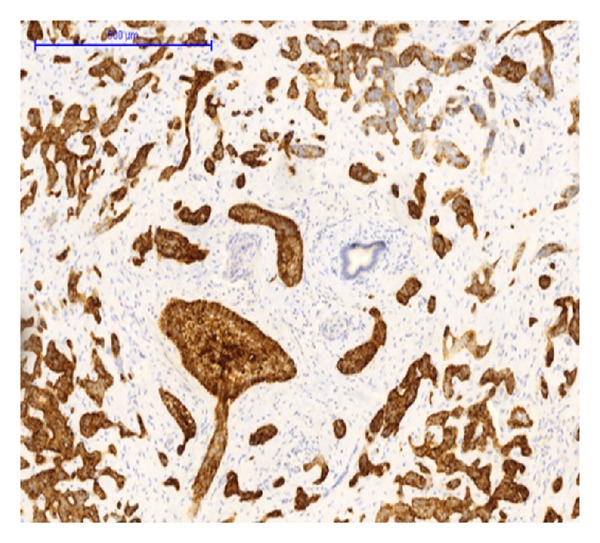
CK5/6 immunohistochemical staining. Hepatic BSCC shows intense staining in the tumor cells, whereas the benign bile duct in the center is negative.

**Figure 8:**
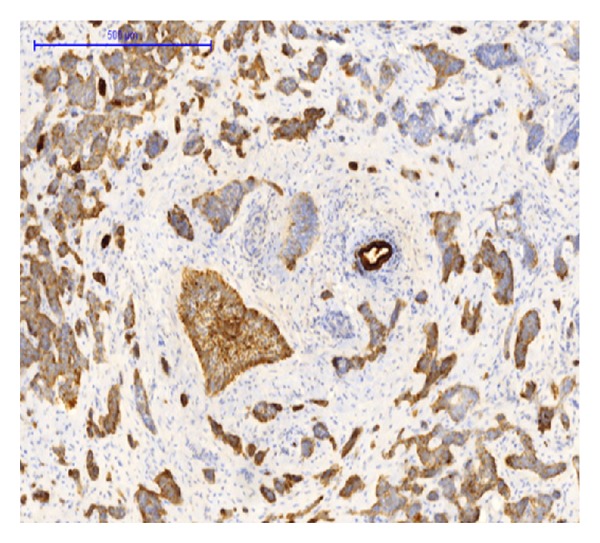
CK7 immunohistochemical staining. The benign bile duct in the center is positive. Some tumor cells show a faint to moderate reaction, whereas other tumor cells are completely negative.

Immunohistochemically the tumor cells stained positive for CK 5/6, EP4, p63, CK7, and CK17 (Figures 6, 7, and 8). A focal, but positive, reaction was seen for EMA and polyclonal CEA, and a few percentage of tumor cells stained positive for chromogranin and CD56. CK20, Cdx2, villin, MUC5AC, MUC2, synaptophysin, vimentin, and glypican were all negative.

Molecular examinations showed no KRAS mutations or mutations in isocitrate dehydrogenase 1 and 2 (IDH 1/2) genes. The surrounding liver tissue showed a preserved lobular architecture. There was no evidence of biliary or hepatic disease.

Based on the morphology and the immunohistochemical findings, the diagnosis of primary basaloid squamous cell carcinoma was made.

## 6. Discussion

Primary squamous cell carcinoma (SCC) of the liver is a rare malignant neoplasm. Only around 26 such cases have been reported in the literature. In this case our primary findings were an asymptomatic patient, who debuted with cholecystitis. After examining the patient we detected tumors in the liver and a small process in the left armpit. Histology revealed a basaloid squamous cell carcinoma (BSCC) in the liver and in an axillary lymph node. Further examinations of the patient showed no other pathological findings, and we, therefore, suspected a primary BSCC of the intrahepatic bile ducts.

The precise mechanism of how SCC develops in the bile ducts is not well described. The leading theory is that SCC usually develops in an environment with chronic inflammation [13]. It has also been proposed to be associated with hepatic teratoma, hepatolithiasis, or solitary, benign, nonparasitic hepatic cyst as reported in previous cases [14–17]. None of these findings were present in our case.

Hepatic teratoma is a neoplasia, usually presenting as a metastatic lesion from an ovarian or testicular primary cancer. These tumors are often cystic and contain a broad variety of cellular components from the different germ layers. In our present case there was no evidence of a primary tumor outside the liver, and the tumor masses were solid and consisted of squamous epithelium without other cellular components.

In cases of hepatolithiasis and benign cysts of nonparasitic origin, metaplasia and eventually dysplasia of the epithelial lining of the bile ducts can occur, giving rise to a primary squamous cell carcinoma of the liver. No signs of hepatolithiasis were found in our case, and no cystic areas were detected in the tumor.

We did find several areas with ductal epithelial transition from squamous metaplastic epithelium to various degrees of dysplasia and invasion, indicating that the tumor could be primary in the liver arising from the intrahepatic bile duct epithelium (Figures 3, 4, and 5). The immunohistochemical profile with CK7, CK5/6, p63, and EMA positivity supports this (Figures 6, 7, and 8). It is however a well known and well described phenomenon to see intraductal tumor spread in both primary and metastatic lesions. And it can be very difficult and sometimes impossible to discriminate whether the ductal lesions represent a dysplastic element or intraductal growth pattern.

A diagnosis of a primary adenosquamous carcinoma should always be considered in cases with squamous morphology, since most cholangiocellular neoplasias are adenomatous. Although numerous tumor tissue blocks were sampled and investigated no glandular areas or signs of mucin production were observed in this present case.

Furthermore, it is well known that primary BSCCs outside the liver can metastasize to the liver [18–20], that is, head- and neck-, anogenital-, esophageal- and lung-BSCCs. Results of a case control study by Soriano et al. even found a 6 times higher risk of distant metastases in BSCC's (including metastasis to the liver) compared to the usual type of squamous cell carcinoma among head and neck cancers [7]. A retrospective review by Lam et al. found a frequency of distant metastasis of 53% among esophageal BSCCs; liver and lungs being the most common sites. In our present case, various clinical and paraclinical examinations revealed no other tumor areas apart from in the liver and the lymph node in the left armpit.

According to WHO classification of tumors in the liver, mutations in the KRAS gene are the most common genetic abnormality in CCs. Concerning cases of ICC, mostly adenocarcinomas, 31% have a mutation in the KRAS gene and 21% have IDH 1 and 2 gene mutation [21, 22]. In our case none of these mutations could be detected.

## 7. Conclusion

In this present case we found a BSCC in the liver and a lymph node metastasis in the armpit. The morphology and immunohistochemical profile of the tumor combined with lack of any other primary tumor rendered the diagnosis of a primary BSCC of the intrahepatic bile ducts. This is to our knowledge the second case report on this very rare entity. Our present case of primary BSCC of the liver ran a very aggressive course with metastasizes to lung, bone, and lymph nodes in spite of surgery and repetitive chemotherapy.

## Figures and Tables

**Table 1:** Antibodies and their corresponding clone, dilution, pretreatment, incubation methods, and source used for immunohistochemical analysis of the biopsies and resected tissue samples.

Antibody	Clone	Dilution	Pretreatment and incubation	Source
CK7	OV-TL 12/30	1 : 1000	1	Dako
CK20	KS20.8	1 : 400	1	Dako
CK17	E3	1 : 100	1	Dako
CK5/6	D5/16	1 : 20	1	Dako
P63	4A4	Ready to use (RTU)	1	Roche
Antihuman epithelial antigen (EP4)	Ber-EP4	1 : 25	2	Dako
Epithelial membrane antigen (EMA)	E29	1 : 50	1	Dako
MUC2	CCP58	1 : 150	1	Dako
MUC5AC	45M1	1 : 500	1	Neomarkers
CDX2	EPR2764Y	RTU	1	Roche
Villin	12D2C3	1 : 40	1	Dako
Polyclonal carcinoembryonic antigen (CEAP)	Poly	1 : 3000	1	Elektrabox
Hepatocyte specific antigen	OCH1E5	1 : 1000	1	Dako
Glypican	GC33	RTU	1	Roche
Vimentin	VIM 3B4	1 : 400	1	Dako
Synaptofysin	Dak-synapt	1 : 1500	1	Dako
Chromogranin	Poly	1 : 2000	1	Dako

Pretreatment and incubation.

(1) HIER pH 8,5/32 min.

(2) Protease 12 min/32 min.

**Table 2:** Primer sequences (For., Rev., Seq.), amplified fragment sizes, and pyrosequencing dispensation and analysis orders (Disp. and Anal.) used for mutational analysis of KRAS, IDH1, and IDH2.

Exon		Primer sequence	Fragment (bp)	Hot spot codons
KRAS				
2	For.	5′-GGCCTGCTGAAAATGACTG	79	12, 13
	Rev.	5′-Biotin-GCTGTATCGTCAAGGCACTCT		
	Seq.	5′-CTTGTGGTAGTTGGAGCT		
	Disp.	TACGACTCAGATCGTAG		
	Anal.	GNTGRCGTAGGYA		
3	For.	5′-CAG ACT GTG TTT CTC CCT TCT CA	133	61
	Rev.	5′-Biotin-TCCTCATGTACTGGTCCCTCATT		
	Seq.	5′-TTG GAT ATT CTC GAC ACA		
	Disp.	TCAGCAGATCGTAGAG		
	Anal.	G/ACAGGTCNAGAGGAGAG		

IDH1				
4	For.	5′-Biotin-TGGATGGGTAAAACCTATCATCA	66	132
	Rev.	5′-TTGCCAACATGACTTACTTGATC		
	Seq.	5′-ATCTGTGAATCCAGAGGGG		
	Disp.	TGCACTGTCACTA		
	Anal.	GAC/TG/AACCTAT		

IDH2				
4	For.	5′-TGTGGAAAAGTCCCAATGGA	176	132
	Rev.	5′-Biotin-AGGTCAGTGGATCCCCTCTC		
	Seq.	5′-GCCCATCACCATTGG		
	Disp.	GCATGCACG		
	Anal.	CAG/A/TGCACGCC		
